# The prognostic value of systemic immune-inflammation index in surgical esophageal cancer patients: An updated meta-analysis

**DOI:** 10.3389/fsurg.2022.922595

**Published:** 2022-08-26

**Authors:** Xiaoqu Li, Shuhao Zhang, Juan Lu, Chao Li, Naibin Li

**Affiliations:** ^1^ Department of Thoracic Surgery, Sichuan GEM Flower Hospital, North Sichuan Medical College, Chengdu, Sichuan, China; ^2^ Rehabilitation Department, Sichuan GEM Flower Hospital, North Sichuan Medical College, Chengdu, Sichuan, China

**Keywords:** systemic immune-inflammation index, surgery, esophageal cancer, prognosis, updated meta-analysis

## Abstract

**Purpose:**

To identify the prognostic role of systemic immune-inflammation index (SII) in esophageal cancer patients receiving operation.

**Methods:**

The PubMed, EMBASE, Web of Science, Cochrane Library, WanFang and CNKI electronic databases were searched up to February 17, 2022 for relevant studies. The hazard ratios (HRs) and 95% confidence intervals (CIs) were combined to assess the association between SII and prognosis in surgical esophageal cancer patients. The primary outcome was overall survival (OS) and secondary outcomes were progression-free survival (PFS) and cancer-specific survival (CSS). All statistical analyses were conducted by STATA 15.0 software.

**Results:**

A total of nine retrospective studies involving 3,565 participates were included. The pooled results indicated that high SII was significantly related with poor OS (HR = 1.58, 95% CI: 1.23–2.02, *P* < 0.001). However, subgroup analysis based on pathological type demonstrated that high SII was an independent predictor for poor OS only in esophageal squamous cell carcinoma (ESCC) patients (HR = 1.72, 95% CI: 1.34–2.21, *P *< 0.001). Besides, SII was also significantly associated with poor PFS (HR = 1.94, 95% CI: 1.61–2.35, *P* < 0.001) and CSS (HR = 1.44, 95% CI: 1.04–1.99, *P* = 0.027) in ESCC patients.

**Conclusion:**

The SII could serve as an independent prognostic factor in surgical ESCC patients and higher SII was related with worse survival. However, more prospective high-quality studies are still needed to verify above findings.

## Introduction

Esophageal cancer is one of the most prevalent malignant tumors with high mortality (1, 2). In Asian countries, squamous cell carcinoma (SCC) accounts for the major pathological type of esophageal cancer. Despite the great development of neoadjuvant chemotherapies and adjuvant chemoradiotherapies, the prognosis of esophageal cancer patients remains poor and surgery is still the most important treatment (3–5). Although the tumor-node-metastasis (TNM) staging system is valuable for the prediction of prognosis and formulation of treatment strategy, patients with the same tumor stage may also experience completely different disease progression. Thus, it is still necessary to identify more valuable prognostic factors for esophageal cancer patients.

In recent years, a lot of studies have demonstrated that systemic inflammation plays an essential role in the incidence and development of cancers (6–9). Furthermore, several inflammatory biomarkers have been manifested to show relatively high prognostic value in esophageal cancer such as the neutrophil to lymphocyte ratio (NLR), lymphocyte to monocyte ratio (LMR), platelet to lymphocyte ratio (PLR), and C-reactive protein to albumin ratio (CAR) (10–14). A novel inflammatory biomarker, systemic inflammation index (SII) which is calculated as (absolute platelet count × absolute neutrophil count)/lymphocyte count, was then established and its high prognostic value has been well identified in several types of cancers such as pancreatic cancer, renal cell carcinoma and gastric cancer (15–17). It is well known that advanced-stage cancer patients are more likely to show abnormal inflammation indexes, which means the prognostic value of SII in early-stage patients who receive the surgery might be limited. Meanwhile, Zhang et al. revealed that the SII might be predive for overall survival (OS) in esophageal cancer based on only five studies in 2019 (18).

Thus, the aim of this updated meta-analysis was to explore the predictive role of SII for long-term survival of esophageal cancer, which might contribute to the clinical management and treatment of esophageal cancer patients.

## Materials and methods

This updated meta-analysis was conducted according to the Preferred Reporting Items for Systematic Reviews and Meta-Analyses guidelines (19).

### Literature search

The PubMed, EMBASE, Web of Science, Cochrane Library, WanFang and CNKI electronic databases were searched up to February 17, 2022 for relevant studies. The following terms were used during the search: “systemic immune-inflammation index”, “SII”, “esophageal”, “esophagus”, “cancer”, “tumor”, “carcinoma”, “neoplasm”, “prognostic”, “prognosis” and “survival”. The MeSH terms and free key words were both used to increase the sensitivity. The detailed search strategy in the PubMed was as follows: (systemic immune-inflammation index OR SII) AND (esophageal OR esophagus) AND (cancer OR tumor OR carcinoma OR neoplasm) AND (prognostic OR prognosis OR survival). Besides, the references cited in included studies were also reviewed.

### Inclusion and exclusion criteria

Inclusion criteria were as follows: (1) patients were pathologically diagnosed with primary esophageal cancer and received the surgery; (2) the SII was calculated as (serum platelet counts*neutrophil counts)/lymphocyte counts; (3) patients were divided into different groups according to the SII and the prognosis was compared between groups; (4) the primary outcome was overall survival (OS) and the secondary outcomes were progression-free survival (PFS) and cancer-specific survival (CSS); (5) the hazard ratios (HRs) and 95% confidence intervals (CIs) for OS, PFS or CSS were reported directly.

Exclusion criteria were as follows: (1) the methodological quality could not be assessed due to the lack of relevant information or low-quality studies with a Newcastle-Ottawa scale (NOS) score of 5 or lower (20); (2) duplicated or overlapped data; (3) meeting abstracts, letters, editorials, case reports and reviews.

### Data extraction and quality assessment

The following information was collected from included studies: the name of first author, publication year, sample size, country, pathological type, tumor-node-metastasis (TNM) stage, cutoff values of SII, source of HR with 95% CI (univariate or multivariate analysis), follow-up interval, pretreatment modality (surgery or neoadjuvant chemoradiotherapy), outcome and HRs with corresponding 95% CIs.

The quality of included studies was assessed according to the NOS score and only high-quality studies with a NOS score of 6 or higher were included in this updated meta-analysis.

The literature search, selection, data extraction and quality assessment were all performed by two authors independently.

### Statistical analysis

All analyses were conducted with STATA (version 15.0; Stata Corporation). The HRs with corresponding 95% CIs were combined to evaluate the association of SII with prognosis in surgical esophageal cancer patients. The Higgins *I^2^* statistic and Cochran's Q test were used to evaluate heterogeneity among studies. Besides, the logHR was pooled using the inverse variance DerSimonian Laired method during the meta-analysis. Significant heterogeneity was defined as *P *< 0.10 and/or *I^2 ^*> 50%, and when significant heterogeneity was observed, the random-effects model was applied; otherwise, the fixed-effects model was applied (21). Sensitivity analysis was conducted to assess the stability of combined results and sources of heterogeneity. Begg's funnel plot and Egger’s linear regression test were performed to evaluate publication bias, and significant publication bias was defined as *P < *0.05 (22). If significant publication bias was observed, then the trim-and-fill method would be performed to identify potentially unpublished studies and their impact on the overall results.

## Results

### Literature research and selection

**Figure 1:**
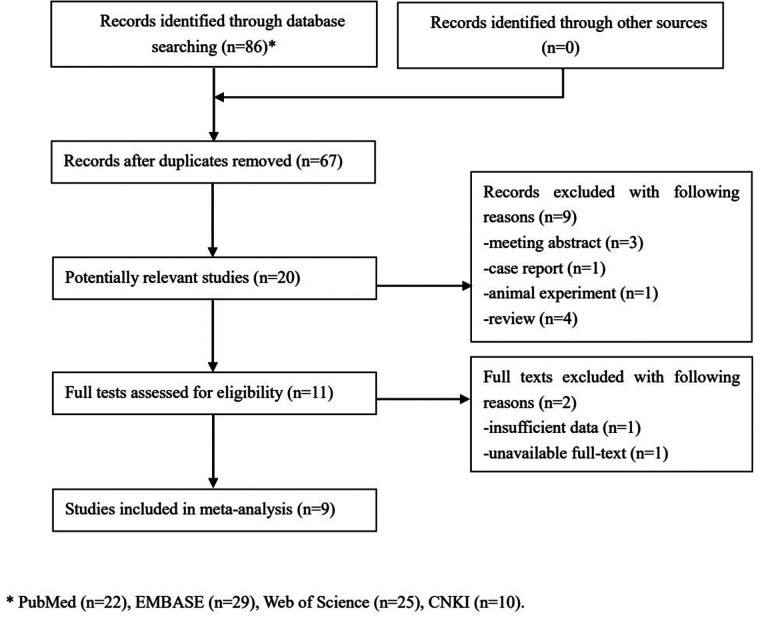
The flow diagram of this meta-analysis.

Initially, 86 records were identified from several databases and 19 duplicated records were removed. Then, 20 potentially relevant publications were further reviewed for eligibility and 11 publications were excluded. Eventually, only nine studies were included in this updated meta-analysis (23–31) (Figure 1).

### Basic characteristics of included studies

All included studies were retrospective and a total of 3,565 patients were enrolled. The sample size and cutoff value of SII ranged from 87 to 916 and from 307 to 792.49, respectively. Most cases were from China and SCC. The other detailed characteristics were presented in Table 1.

**Table 1:** Basic characteristics of included studies.

Author	Year	Sample size	Country	Pathological type	TNM	Cutoff value	Source of HR	Follow-up interval	Pretreatment modality	Outcome	NOS
Geng (23)	2016	916	China	SCC	I-III	307	M	3–146 months	Surgery	OS	8
Feng (24)	2017	298	China	SCC	I-III	410	M	1–101 months	Surgery	CSS	7
Wang (25)	2017	280	China	SCC	I-IV	560	M	1–48 months	Surgery	OS, PFS	7
Ishibashi (26)	2018	143	Japan	EC	I-IV	650	M	NR	Surgery	OS	7
Zhang (28)	2018	655	China	SCC	I-III	387.65	M	3–144 months	Surgery	OS	7
Gao (27)	2019	468	China	SCC	I-III	479.72	M	3.2–114.5 months	Surgery	OS, PFS	7
Cai (29)	2020	311	China	SCC	II-III	583.45	M	22 (median)	Neoadjuvant chemoradiotherapy	OS, PFS	6
Zhao (30)	2020	87	China	SCC	II-III	792.49	M	9.6–77.4 months	Neoadjuvant chemoradiotherapy	OS	6
Qi (31)	2021	407	China	EC	I-IV	433.25	U	29 (median)	Surgery	OS	6

CSS, cancer-specific survival; EC, esophageal cancer; M, multivariate analysis; NOS, Newcastle Ottawa Scale; NR, not reported; OS, overall survival; PFS, progression-free survival; SCC, squamous cell carcinoma; U, univariate analysis.

### The association between SII and OS of esophageal cancer patients

**Figure 2:**
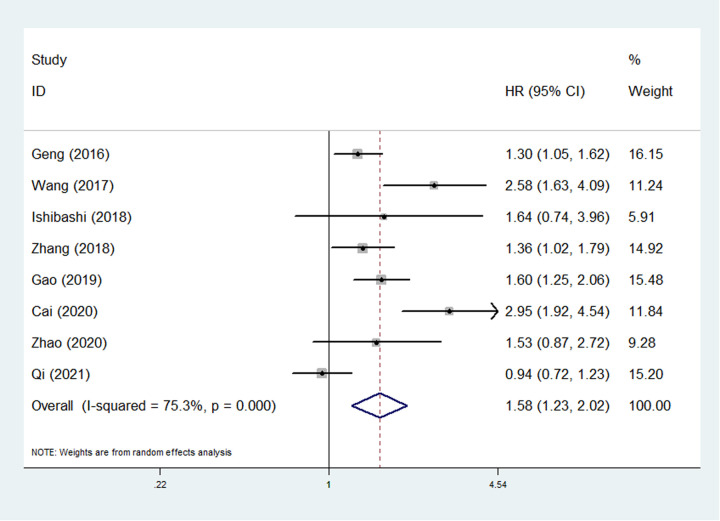
The association between systemic inflammation index and overall survival in esophageal cancer.

Eight studies involving 3,267 patients explored the relationship between SII and OS (23, 25–31). The pooled results demonstrated that SII was an independent predictor for OS (HR = 1.58, 95% CI: 1.23–2.02, *P* < 0.001; *I*^2 ^= 75.3%, *P *< 0.001) (Figure 2). Then the subgroup analysis based on the pathological type was conducted and the results showed that high SII was only significantly associated with poor OS of esophageal SCC (ESCC) patients (HR = 1.72, 95% CI: 1.34–2.21, *P *< 0.001; *I*^2 ^= 70.0%, *P* = 0.005) (Table 2).

**Table 2:** Results of meta-analysis.

	No. of studies	HR	95% CI	*P* value	*I*^2^[%]	*P* value
Overall survival	8	1.58	1.23–2.02	<0.001	75.3	<0.001
Pathological type						
Squamous cell carcinoma	6	1.72	1.34–2.21	<0.001	70.0	0.005
Esophageal cancer	2	1.07	0.68–1.70	0.759	34.3	0.217
Progression-free survival	3	1.94	1.61–2.35	<0.001	44.2	0.167
Cancer-specific survival	1	1.44	1.04–1.99	0.027	–	–

HR, hazard ratio; CI, confidence interval.

### The association between SII and PFS and CSS of esophageal cancer patients

**Figure 3:**
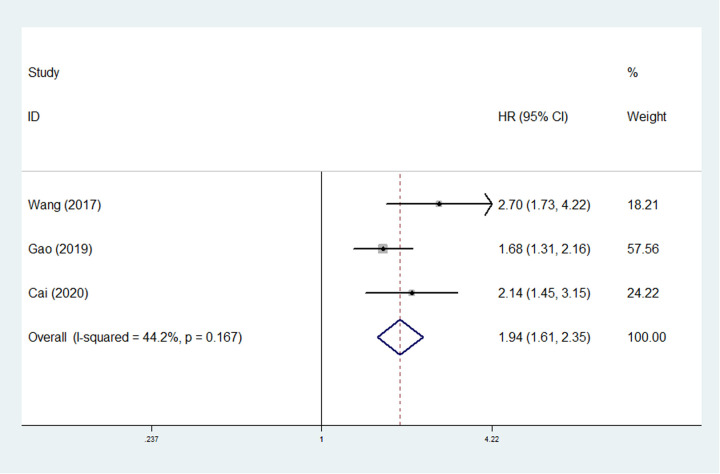
The association between systemic inflammation index and progression-free survival in esophageal cancer.

Only three studies involving 1,357 surgical ESCC patients explored the relationship between SII and PFS (25, 27, 29). The pooled results manifested that higher SII was an independent predictor for worse PFS (HR = 1.94, 95% CI: 1.61–2.35, *P* < 0.001; *I*^2 ^= 44.2%, *P* = 0.167) (Figure 3) of ESCC patients.

Only Feng et al. reported the predictive role of SII for CSS in esophageal cancer (ESCC) patients and a positive relationship between SII and CSS was presented (HR = 1.44, 95% CI: 1.04–1.99, *P* = 0.027).

### Sensitivity analysis and publication bias

**Figure 4:**
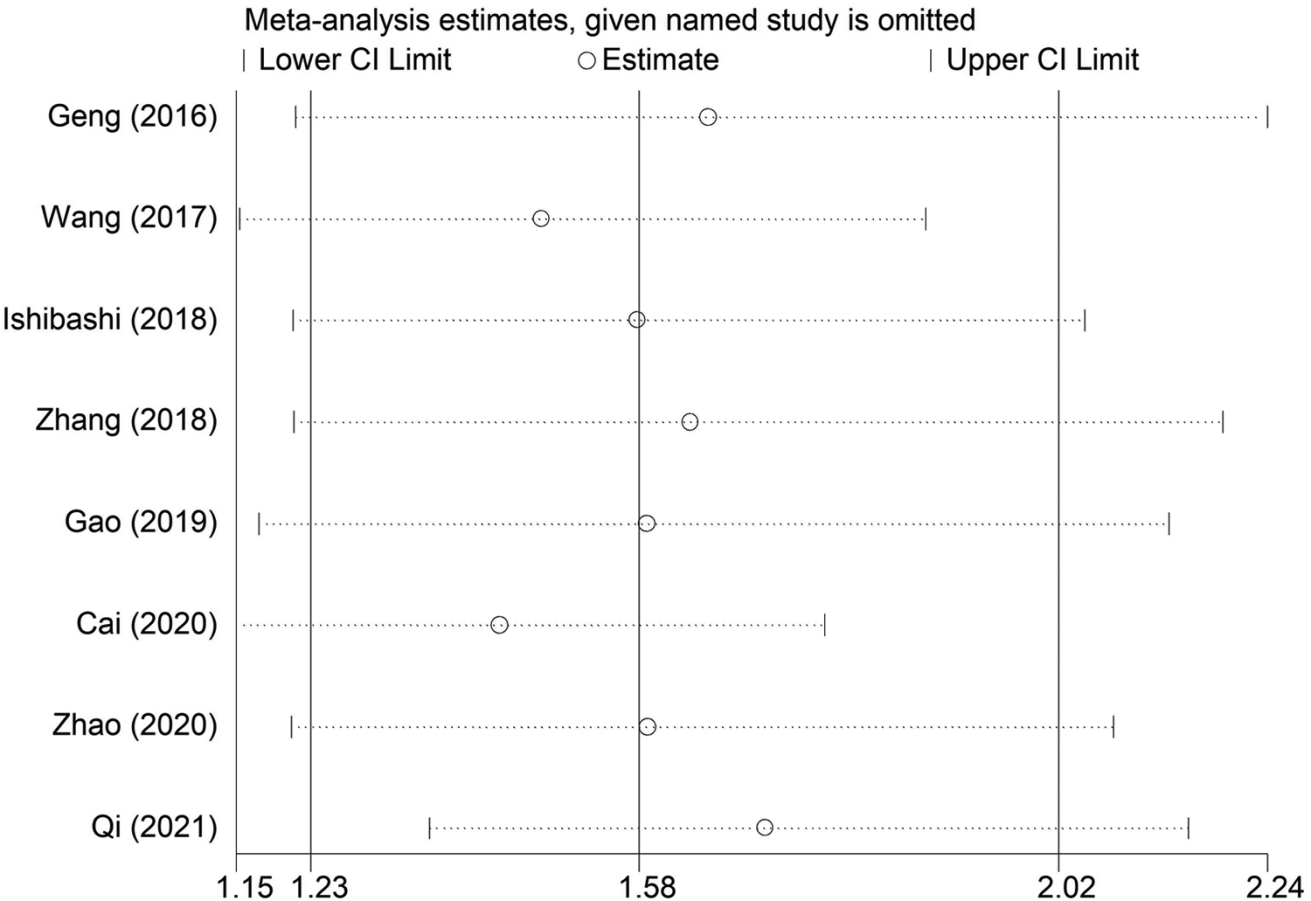
Leave-one-out analysis about the association between systemic inflammation index and overall survival in esophageal cancer.

**Figure 5:**
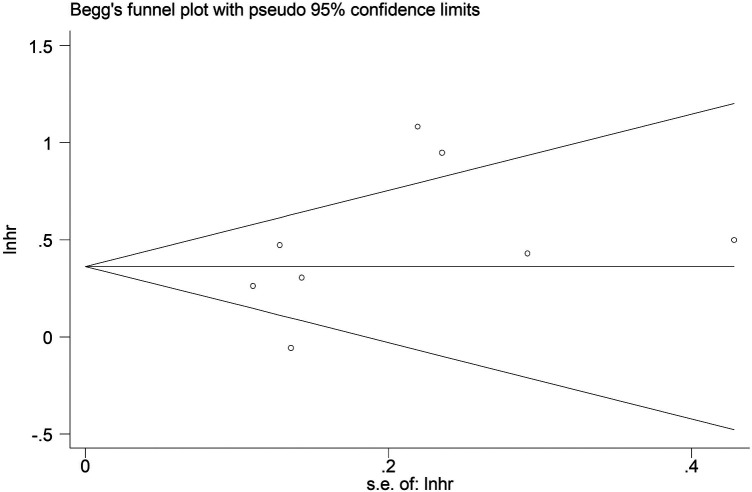
Begg’s funnel plot.

The sensitivity analysis revealed that the pooled results were stable and reliable (Figure 4). Furthermore, no obvious publication bias was observed in this updated meta-analysis according to the symmetric Begg’s funnel plot (Figure 5) and *P* = 0.199 of Egger’s test.

## Discussion

This updated meta-analysis demonstrated that SII was an independent prognostic risk factor for surgical ESCC patients and higher SII predicted worse survival after including nine retrospective studies involving 3,565 patients. Thus, the SII could contribute to the evaluation of long-term survival and formulation of treatment strategies for operated ESCC patients.

Although a great number of studies have verified that systemic inflammation plays an essential role in the development and progression of esophageal carcinoma, the specific mechanisms are still not very clear. There are several possible explanations for the close association of systemic inflammation with poor survival of cancer patients. First, platelets could directly interact with cancer cells and release some cytokines which play a role in promoting tumor growth, invasion, and angiogenesis (32). Besides, platelets also contribute to the metastasis by stimulating cancer cell proliferation, stabilizing cancer cell arrest in vasculatures, and enhance tumor cell extravasation (33). Second, neutrophils also play a role in promoting tumor cell proliferation by secreting some proteolytic enzymes and serine proteases, stimulating tumor angiogenesis by secreting some proangiogenic factors like the vascular endothelial growth factor (VEGF), and inducing local immunosuppression through impairing T-cell responses and inducing T-cell death (34). Third, it has been widely manifested that T-lymphocytes could effectively inhibit cancer cell proliferation and metastasis, induce cytotoxic cell death and promote anti-tumor immune responses (35). In addition to above mentioned, systemic inflammation is also closely related to the treatment responses of esophageal cancer.

The prognostic value of SII has been identified in several cancers. Wang et al. included nine studies involving 2,441 participantes and demonstrated that elevated SII was an independent predictor for worse OS (HR = 1.88, *P* < 0.001), CSS (HR = 1.852, *P* = 0.007), and PFS/disease-free survival (DFS) (HR = 2.50, *P* = 0.014) in non-small cell lung cancer (NSCLC) (36). Besides, Li et al. revealed that elevated SII was significantly associated with poorer OS (HR = 1.55, *P* < 0.001) and CSS/PFS/DFS (HR = 1.51, *P* < 0.001) after reviewing 2,132 pancreatic cancer patients from seven studies (17). Furthermore, after including 11 relevant studies Fu et al. manifested that SII was significantly related to OS (HR = 1.53, 95% CI: 1.27–1.83) and DFS (HR = 1.57, 95% CI: 1.24–1.97) in gastric cancer (15). The results of this meta-analysis are consistent with previous findings.

There are still some valuable fields about the SII in esophageal cancer worth more investigation. Most esophageal cancer patients receive non-surgical therapies such as neoadjuvant chemotherapies and postoperative adjuvant chemotherapies. Thus, it is necessary to explore the clinical role of SII in predicting the treatment responses in esophageal cancer patients. Besides, most studies only focused on the clinical role of pretreatment SII. However, the dynamic change of SII may be more useful for prediction of prognosis and development of treatment strategies. All patients are from Asian countries (China or Japan) and most cases are ESCC in this meta-analysis. The prognostic value of SII in other countries and populations remains unclear.

There were several limitations in this study. First, all studies were retrospective and from Asian countries, which might cause some bias. Second, we were unable to conduct more subgroup analysis based on other important parameters such as the TNM stage, age and sex due to the lack of detailed data. Third, the cutoff values of SII ranged from 307 to 792.49, but it was unable to determine the optimal cutoff values of SII in predicting prognosis of surgical esophageal cancer patients. Four, in the subgroup analysis stratified by the pathological type of our meta-analysis, no significant association between SII and esophageal cancer was observed based on two relevant studies. Thus, more studies are needed to further identify the prognostic value of SII in esophageal cancer.

## Conclusion

The results of this study demonstrated that SII could serve as an independent prognostic factor in surgical ESCC patients and elevated SII was related with worse survival. However, more prospective high-quality studies are still needed to verify above findings.

## Data Availability

The original contributions presented in the study are included in the article/Supplementary Material, further inquiries can be directed to the corresponding author/s.
